# The first hominin from the early Pleistocene paleocave of Haasgat, South Africa

**DOI:** 10.7717/peerj.2024

**Published:** 2016-05-11

**Authors:** AB Leece, Anthony D.T. Kegley, Rodrigo S. Lacruz, Andy I.R. Herries, Jason Hemingway, Lazarus Kgasi, Stephany Potze, Justin W. Adams

**Affiliations:** 1 The Australian Archaeomagnetism Laboratory, Department of Archaeology and History, La Trobe University, Bundoora, Victoria, Australia; 2 Department of Anatomy and Developmental Biology, Monash University, Melbourne, Victoria, Australia; 3 Department of Biomedical Sciences, Grand Valley State University, Allendale, MI, United States of America; 4 Department of Basic Science and Craniofacial Biology, New York University, New York, NY, United States of America; 5 Centre for Anthropological Research, University of Johannesburg, Johannesburg, Gauteng, South Africa; 6 School of Anatomical Sciences, Faculty of Health Sciences, University of the Witwatersrand, Johannesburg, South Africa; 7 Plio-Pleistocene Section, Department of Vertebrates, Ditsong National Museum of Natural History, Pretoria, South Africa

**Keywords:** *Paranthropus*, *Australopithecus*, *Homo*, Paleomagnetism, Paleokarst, Enamel microstructure

## Abstract

Haasgat is a primate-rich fossil locality in the northeastern part of the Fossil Hominid Sites of South Africa UNESCO World Heritage Site. Here we report the first hominin identified from Haasgat, a partial maxillary molar (HGT 500), that was recovered from an *ex situ* calcified sediment block sampled from the locality. The *in situ* fossil bearing deposits of the Haasgat paleokarstic deposits are estimated to date to slightly older than 1.95 Ma based on magnetobiostratigraphy. This places the hominin specimen at a critical time period in South Africa that marks the last occurrence of *Australopithecus* around 1.98 Ma and the first evidence of *Paranthropus* and *Homo* in the region between ∼2.0 and 1.8 Ma. A comprehensive morphological evaluation of the Haasgat hominin molar was conducted against the current South African catalogue of hominin dental remains and imaging analyses using micro-CT, electron and confocal microscopy. The preserved occlusal morphology is most similar to *Australopithecus africanus* or early *Homo* specimens but different from *Paranthropus*. Occlusal linear enamel thickness measured from micro-CT scans provides an average of ∼2.0 mm consistent with *Australopithecus* and early *Homo*. Analysis of the enamel microstructure suggests an estimated periodicity of 7–9 days. Hunter–Schreger bands appear long and straight as in some *Paranthropus*, but contrast with this genus in the short shape of the striae of Retzius. Taken together, these data suggests that the maxillary fragment recovered from Haasgat best fits within the *Australopithecus*—early *Homo* hypodigms to the exclusion of the genus *Paranthropus*. At ∼1.95 Ma this specimen would either represent another example of late occurring *Australopithecus* or one of the earliest examples of *Homo* in the region. While the identification of this first hominin specimen from Haasgat is not unexpected given the composition of other South African penecontemporaneous site deposits, it represents one of the few hominin localities in the topographically-distinct northern World Heritage Site. When coupled with the substantial differences in the mammalian faunal communities between the northern localities (e.g., Haasgat, Gondolin) and well-sampled Bloubank Valley sites (e.g., Sterkfontein, Swartkrans, Kromdraai), the recovery of the HGT 500 specimen highlights the potential for further research at the Haasgat locality for understanding the distribution and interactions of hominin populations across the landscape, ecosystems and fossil mammalian communities of early Pleistocene South Africa. Such contextual data from sites like Haasgat is critical for understanding the transition in hominin representation at ∼2 Ma sites in the region from *Australopithecus* to *Paranthropus* and early *Homo*.

## Introduction

The Haasgat fossil site is a paleokarstic deposit consisting of a large cave passage completely in-filled with calcified cave sediments and interstratified speleothem, the latter of which was largely removed by lime mining at the turn of the 20th century. This mining activity also generated a large talus slope outside the cave consisting of fossil bearing paleocaves sediment blocks that have yielded the Haasgat *ex situ* faunal assemblage (HGD; Keyser, 1991; Keyser & Martini, 1991; McKee & Keyser, 1994; Plug & Keyser, 1994; Adams, 2012). The Haasgat primate community is unique relative to that reported from most Bloubank Valley sites (e.g., Sterkfontein, Swartkrans, Cooper’s, Kromdraai), with the most demographically diverse and potentially oldest sample of the basal *Papio* species, *Papio angusticeps,* in the African fossil record (Freedman, 1957; Gilbert et al., 2015). Haasgat is also the only site currently documenting multiple fossil colobine species (*Cercopithecoides haasgati, Cercopithecoides williamsi, Cercopithecoides* sp.) in South Africa (McKee & Keyser, 1994; McKee, Von Mayer & Kuykendall, 2011; Kegley, Hemingway & Adams, 2011; Adams, 2012; Adams et al., 2015). The overall mammalian faunal assemblage is also unusual in the low representation of Order Carnivora, as well as the recovery of primitive alcelaphins/ovibovins and a high proportion of extremely large klipspringers (*Oreotragus* sp.) that are more similar to those from the Makapansgat Member 3 deposits (3.03–2.58 Ma; Herries et al, 2013) than those recovered from the nearby Gondolin GD 2 deposits (∼1.8 Ma; Herries et al., 2006; Adams, 2010; Adams, 2012). Despite this rich and regionally-unique faunal community established through the *ex situ* HGD assemblage, no hominins were recovered during the first phases of paleontological sampling at Haasgat. This paper provides a comprehensive morphological and dental microstructure analysis of the first hominin specimen, a partial maxillary molar (HGT 500; Fig. 2), that was recovered from a calcified *ex situ* dumpsite block collected in 2011 from the site.

**Figure 1:**
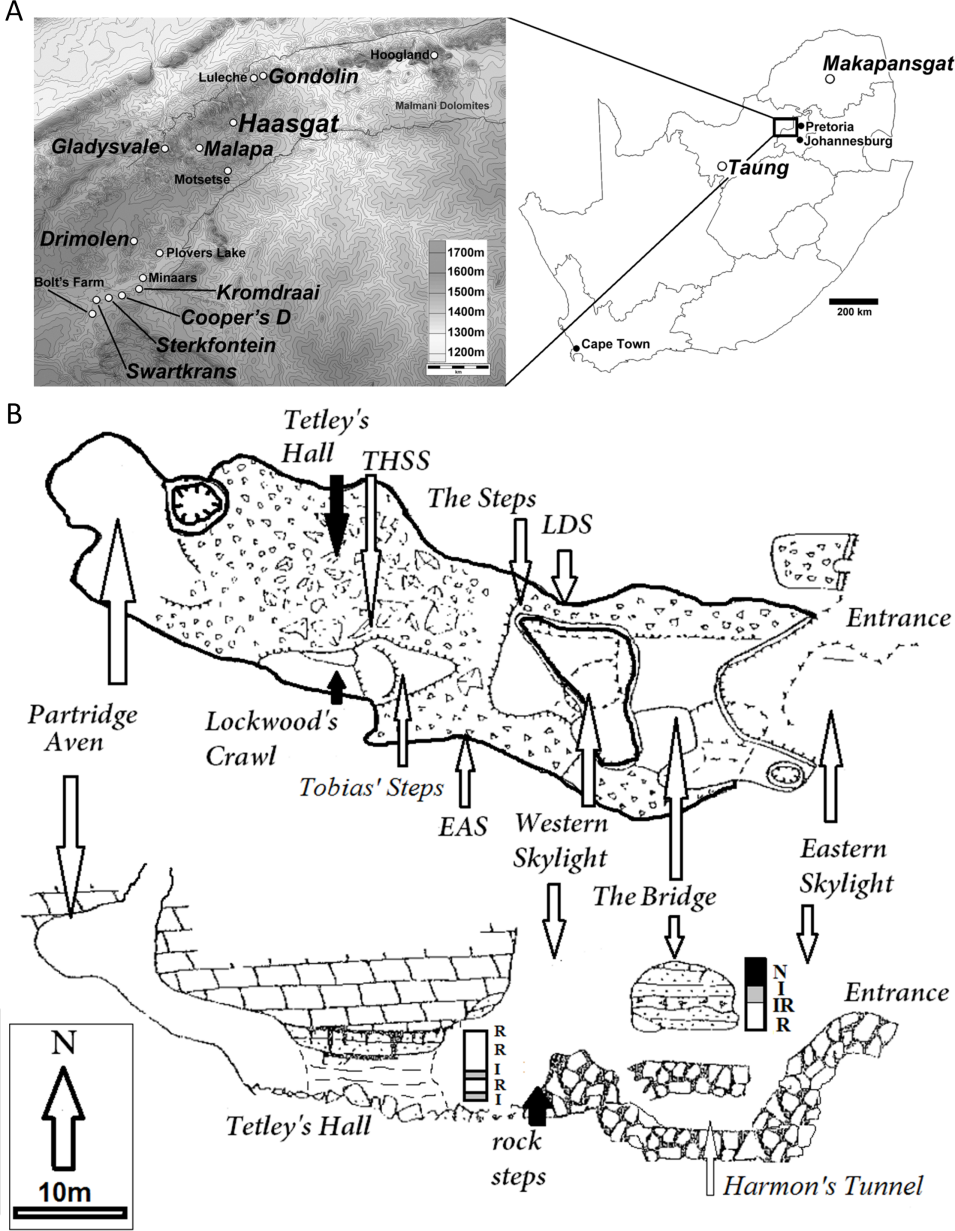
Location and site plan of the Haasgat paleocave system. (A) topographic contour map indicating the position of Haasgat relative to other South African late Pliocene and early Pleistocene sites and location of these sites within South Africa. (B) plan and section views of the excavated Haasgat paleocave system. Scale bars as indicated.

**Figure 2:**
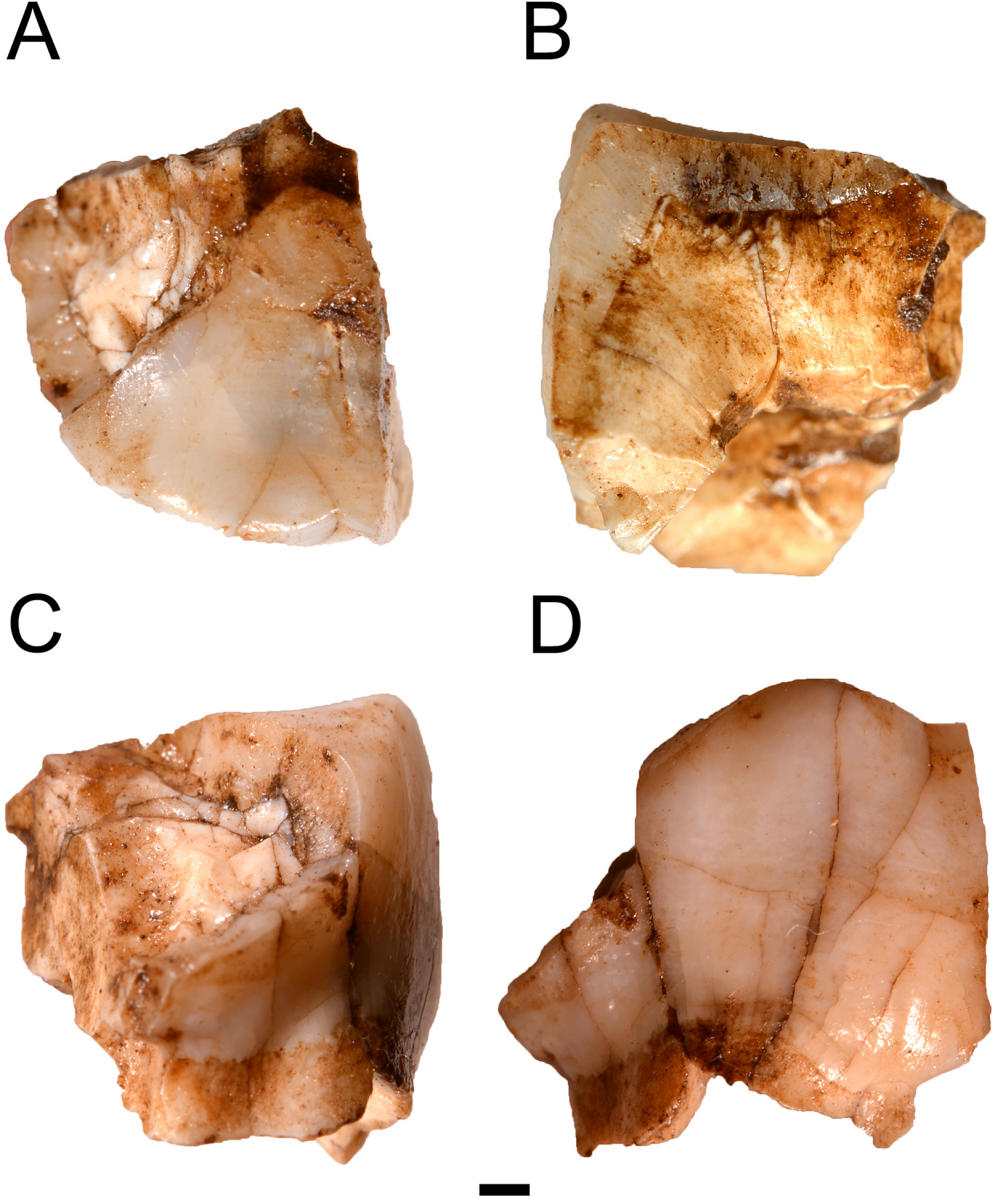
The HGT 500 Hominini gen. et sp. indet. molar. (A) occlusal view; (B) mesial view; (C) distal view; (D) buccal view. Scale bar equals 1 mm.

The unique nature of the fossil assemblage may also reflect the fact that the site is formed within the Eccles Formation of the Malmani dolomite in the northern sector of the Hominid Fossil Sites of South Africa UNESCO World Heritage Site (known locally as the Cradle of Humankind or ‘Cradle’; Fig. 1: ∼50 km north-northwest of Johannesburg in Gauteng Province). This high topographic setting, with upland plateaus and deeply incised valleys is quite distinct from the southern area of the Cradle and the Bloubank Valley, from which the majority of the Plio-Pleistocene paleontological data has been recovered (Sterkfontein, Swartkrans, Kromdraai, Cooper’s). This landscape is indicative of highly variable erosional processes during the Neogene and reflected in the variable paleohabitats and mammalian community composition between sites across the region (Vrba, 1976; Brain, 1981; Reed, 1996; Avery, 2001; Adams & Conroy, 2005; Adams et al., 2010; Bailey, Reynolds & King, 2011; Adams, 2012; Adams, Kegley & Krigbaum, 2013). These differences are further reflected in reconstructions that suggest the local environments during deposition were both mesic and rugged as evidenced by trace geochemical analysis (Herries et al., 2014) and the dominance of C3-consuming and/or broken terrain adapted species in the *ex situ* HGD faunal assemblage (Adams, Kegley & Krigbaum, 2013).

Unlike the Bloubank Valley caves, Haasgat does not suffer from major phases of karstification that have caused the mixing of fossils from different aged deposits (Reynolds, Clarke & Kuman, 2007; Herries & Adams, 2013). While two phases of cave formation occurred at Haasgat, the later sediment fill is distinct (uncalcified, greyish-brown) from the older (calcified, reddish-brown) paleocave sediments (Herries & Adams, 2013; Herries et al., 2014). Given similarities with *in situ* excavated material, the *ex situ* derived fauna is considered a reliable temporal indicator of both the *ex situ* and *in situ* fossil assemblages. Only recently have comprehensive geological, geochemical and paleobiological analyses been undertaken alongside the first excavations into the rich *in situ* sediment deposits (Kegley, Hemingway & Adams, 2011; Adams, 2012; Adams, Kegley & Krigbaum, 2013; Herries et al., 2014; Adams et al., 2015; J Adams, 2016, unpublished data). The recent recovery of *P. angusticeps, C. haasgati*, and *C. williamsi* craniodental remains from the first *in situ* excavations (Adams et al., 2015; J Adams, 2016, unpublished data) confirms data from the *ex situ* assemblage and has also established the occurrence of partial skeletons within the deposits. These *in situ* sediments have been dated to slightly older than 1.95 Ma based on the occurrence of *in situ Equus* material (thus <2.3 Ma) within the walls of the deposit as well as a magnetic reversal correlated to the base of the Olduvai SubChron in the upper portion of the sediment sequence (Herries et al., 2014). As such, the site covers a critical time period in the South Africa fossil record with the extinction of *Australopithecus* by ∼2 Ma (*Au. africanus* at the end of Sterkfontein Member 4 deposition and *Au. sediba* from Malapa) and the first occurrence of *Paranthropus*, early *Homo*, and the first mode 1 stone tools by 1.8 Ma (Herries et al., 2010; Herries & Shaw, 2011; Pickering et al., 2011a; Pickering et al., 2011b; Herries & Adams, 2013).

**Table 1:** South African hominin specimens used in comparative analysis of HGT 500.

Species	Locality	Specimen
*Paranthropus robustus*	Drimolen	DNH 44, 47, 84, 107, 108
	Kromdraai B	KB 5222, 5383, TM 1536, 1517, 1601c, 1603
	Swartkrans Member 1HR	SK 13, 14, 31, 36, 41, 42, 46, 47, 48, 52, 57, 62, 83, 98, 102, 829, 831, 832, 833, 834, 835, 836, 838, 849, 870, 872, 3975, 3977, 25605
		SKW 11, 14, 3114
*Australopithecus africanus*	Sterkfontein Member 4 (Type Site)	STS 1, 2, 8, 19, 12, 17, 19^a^, 21, 22, 23, 24, 32,
		37, 43, 52, 53, 54, 57, 71, 72, 1881, 3009
		TM 1512, 1561
	Sterkfontein Member 5c (West Pit)	SE 255, 1508
	Makapansgat Limeworks	MLD 11/30
	Sterkfontein	STW 151^b^
*Australopithecus sediba*	Malapa	MH 1
*Homo erectus (sensu lato)*	Swartkrans Member 1HR	SK 27
South African Early *Homo*	Drimolen	DNH 35, 67/70/71, 83
	Swartkrans Member 1HR	SK 874
	Swartkrans Member 2	SKX 267
	Kromdraai B	KB 5223^c^

**Notes.**

These materials are curated at the Ditsong National Museum of Natural History in Pretoria, South Africa and at the Evolutionary Studies Institute, University of Witwatersrand in Johannesburg, South Africa (Table 1).

a Ahern (1998) and Lockwood & Tobias (2002); Argued to represent early *Homo* by Kimbel & Rak (1993).

b Unknown provenience; Recently published as “Indeterminate Hominin” (Smith et al., 2015).

c Originally assigned to *Paranthropus* (Grine, 1982) but recently ascribed to *Homo* (Braga & Thackeray, 2003).

**Figure 3:**
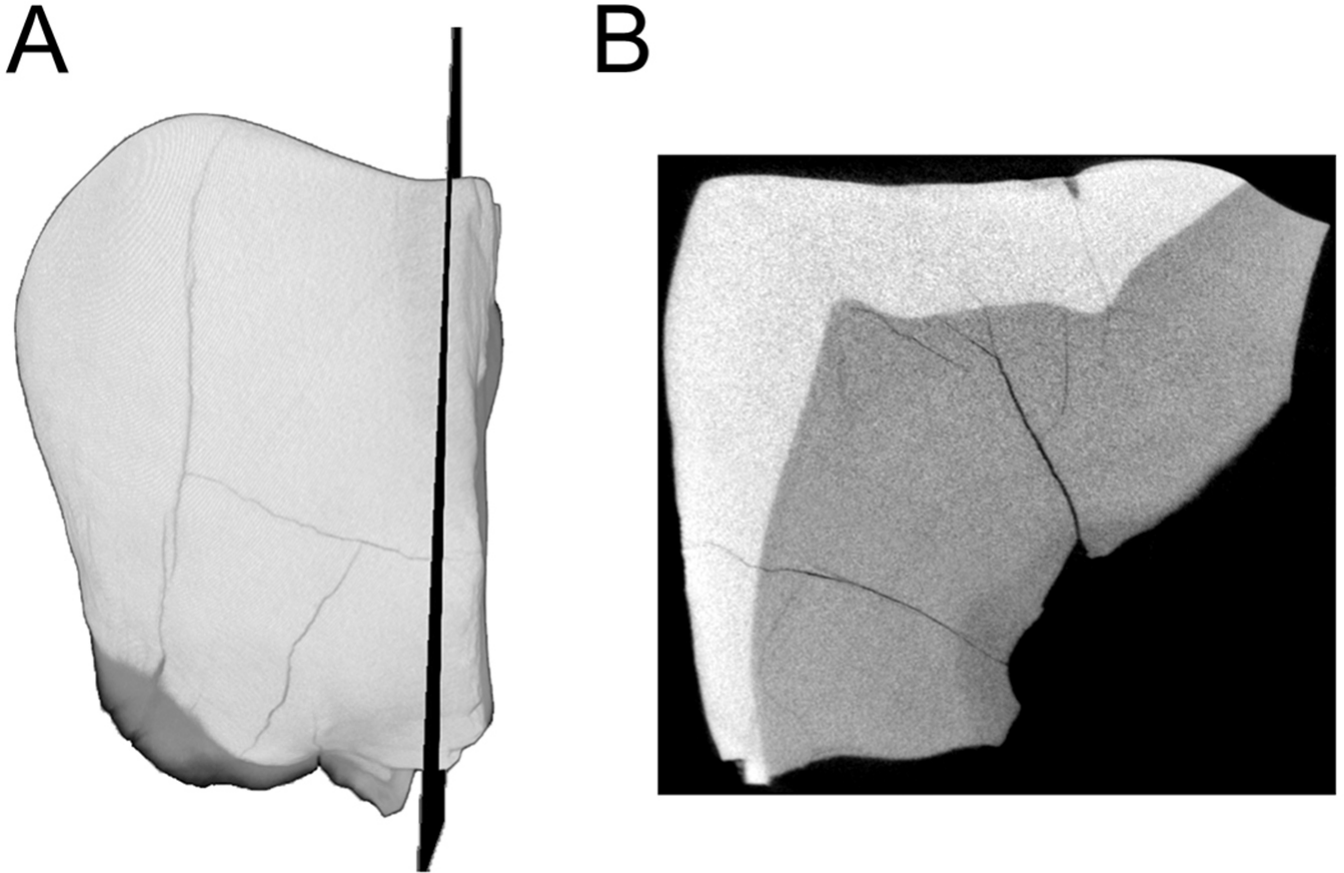
MicroCT derived surface render of the HGT 500. (A) mesial view of the specimen demonstrating the section plane used for evaluating enamel thickness. (B) section plane through the molar demonstrating enamel thickness and morphology of the EDJ and dentin horns.

## Materials and Methods

The HGT 500 specimen was processed from an *ex situ* block recovered from the calcified sediment dumpsite in 2011. The original ∼10 cm × ∼25 cm reddish siltstone calcified block (HGT 11-50) is consistent with deposits occurring in the reversed polarity deposits at the rear of the paleocave system. The block was processed using a 5% acetic acid solution and stabilized with the thermoplastic resin Paraloid™ B-72 (Dow Chemical Co., USA) following protocols of the fossil preparation lab at the Plio-Pleistocene Section, Ditsong National Museum of Natural History, Pretoria (the repository institution of Haasgat fossil specimens collected since 2009; all assigned a ‘HGT’ prefix to identify them relative to the previously collected, *ex situ* HGD fossils from the site curated with the Council for Geosciences, Pretoria). The HGT 500 specimen is one of only three identifiable macromammalian craniodental specimens recovered from the block, with the other two representing an indeterminate Class II (*sensu* Brain, 1981: Table 1) bovid central cavity (Family Bovidae, Order Cetartiodactyla; HGT 557) and a partial hyrax (*Procavia* sp.; Family Procaviidae, Order Hyracoidea) molar (HGT 558). One partial right coracoid (HGT 554) and one right distal humerus (HGT 560) are likely derived from a single indeterminate avian. The remaining 58 fossils recovered from the block consist of: six <1 cm indeterminate taxon enamel fragments, one partial indeterminate epiphysis, and indeterminate element diaphyseal fragments (1–2 cm: 32, 2–3 cm: 17, 3–4 cm: 2). All assessable elements record minimal evidence of cortical exfoliation from environmental exposure (Weathering Stage 0: 7, Stage 1: 9, Stage 2: 3; *sensu* Behrensmeyer, 1978), porcupine gnawing is evident on at least two indeterminate diaphyseal specimens (HGT 568 and HGT 576; possibly HGT 756), and possible carnivore-induced gnawing on two elements (HGT 568 and HGT 575). Although a further 24 fossiliferous *ex situ* calcified blocks were collected from across the exposed surface of the HGD dumpsite during the same field season, processing of these blocks did not yield fossil specimens that can be confidently associated with those from HGT 11-50 (J Adams, 2016, unpublished data).

### Direct morphological comparison

The specimens used in comparative analysis included craniodental remains attributed to *Paranthropus robustus, Australopithecus africanus, Australopithecus sediba,* South African early *Homo*, and *Homo habilis* (Table 1).

### Specimen imaging

#### MicroCT

Preparation of the HGT 500 for analysis included removal of residual Paraloid™ , which allowed a naturally-fractured section of the crown to separate (this section was rejoined after scanning was completed). This larger portion of HGT 500 was scanned and reconstructed on a SCANCO microCT 50 (SCANCO Medical, Brüttisellen, Switzerland) housed at the Institute for Genetic Medicine, University of Southern California. Acquisition settings were: 70 kVp, 200 uA, 750 ms exposure, 750 projections, 10 micron isotropic resolution (FOV: 15.1 × 15.1 × 10.58 mm, Grid: 1,510 × 1,510 × 1,058). The resulting microCT TIFF stack is openly available for download through Morphosource (10.17602/M2/M8952) as part of the Ditsong National Museum of Natural History Digital Archive (Adams et al., 2015). Images were converted to DICOM format (NEMA Standard PS3) and imported into AMIRA^©^ 5.4.0 (FEI™ Visualization Sciences Group, Hillsboro, OR, USA) for volume/surface rendering and measurement. Linear measurements (in millimetres) of enamel thickness were taken from the enamel dentine-junction to the outer-most enamel surface from virtual sections in various planes following a similar methodology to Grine & Martin (1988). Given the wear on this specimen, the linear measurements are not exactly taken at the true occlusal plane (to the cusp tip). No attempts were made to estimate the loss of enamel. Instead, the thickness represents a point between the occlusal and lateral enamel, also recorded by Grine & Martin (1988) in the specimens described therein. Virtual cross-sections were aligned perpendicular to a horizontal plane using the cemento-enamel junction as a reference and passing near the dentine horn (Fig. 3A).

#### Portable confocal microscopy

A portable confocal scanning optical microscope was used to analyze the enamel microstructure using 10 × and 20 × objective lenses following previously described methods (Bromage et al., 2007; Lacruz et al., 2008).

## Results

### Comparative morphological description

The HGT 500 specimen is a partial molar preserving one complete cusp, small portions of two additional cusps, and very minimal root structures below the cemento-enamel junction (Fig. 2). Due to the incomplete nature of this specimen, crown and root measurements could not be taken for the purposes of comparison. The overall occlusal shape of the remaining cusp is indicative of a left maxillary molar, as it does not demonstrate the smaller, more closely-packed cusps seen in mandibular molars (Grine, 1984).

Following this positioning the preserved cusps represent a complete metacone and portions of the paracone, protocone, and metaconule. The metacone is complete from the cervical margin to the occlusal margin and the premetacrista and postmetacrista through the trigone basin are visible on the occlusal surface. The paracone is broken along the coronal plane with only a distal portion preserved showing the postparacrista from the cervical margin to the trigone basin. The mediodistal portion of the protocone is preserved at the trigone basin. The buccal-most portion of the metaconule is preserved along the cervical margin and does not reach the occlusal surface. The crown displays a significant flaring at the cemento-enamel junction on the distal face while the buccal face is very straight, running vertically to the occlusal surface. The lack of bulbous lateral faces is distinct from the morphological suite observed in *Paranthropus robustus* and is more suggestive of *Australopithecus* or *Homo*. Minor torsion in the preserved state of this smaller fragment makes it appear to protrude further than it would have prior to deposition.

The occlusal surface preserves a portion of a dentine exposure. It has been estimated that this exposure would have occupied the majority of the absent protocone. This level of wear indicates moderate to heavy attrition. A shallow groove is visible between the metacone and remaining paracone. This is distinctly different from the deeply incised buccal groove seen in *P. robustus* and is instead more similar to those seen in both *Australopithecus africanus* and *Australopithecus sediba* (Grine, 1984; Berger et al., 2010).

The lack of a distal interproximal facet on the preserved surface of this specimen may indicate that it represents either an M^3^ or an M^2^ lacking this facet with the M^3^ not yet in occlusion. The absence of this feature may eliminate the possibility of the specimen representing an M^1^ given that the advanced wear would imply that the M^2^ would have been in occlusion. Alternatively, it is possible the portion of the distal face expected to show an interproximal wear facet has not preserved.

The linear enamel thickness measured along three planes of the larger fragment provided an average of 2.03 mm (range 1.99 mm–2.13 mm) in the occlusal area (Fig. 3). This linear enamel thickness average is inconsistent with values previously reported for *P. robustus* M^2^, and instead falls within the upper boundary of enamel thickness for early South African *Homo* M^2^ (Table 2). However, if HGT 500 represents either an M^1^ or an M^3^, it would result in an enamel thickness outside the range of early South African *Homo* and instead within the range of *A. africanus* (Table 2).

**Table 2:** Enamel thickness of South African hominin species by element.

Species	M1 Mean	M1 Min-Max	M2 Mean	M2 Min-Max	M3 Mean	M3 Min-Max	Species Mean
*Australopithecus africanus*^a^							1.63
Max	1.59	−	−	−	1.67	−	
Mand	1.68	1.31–2.05	1.36	1.23–1.49	1.33	0.98–1.60	
*Paranthropus robustus*^a^							2.03
Max	2.04	1.68–2.39	−	−	1.78	1.37–2.03	
Mand	1.85	1.35–2.33	1.56	−			
Early *Homo*^b^							−
Max	−	−	2.2	−	−	−	
Mand	1.77	−	−	−	−	−	

**Notes.**

a Data from Olejniczak et al. (2008).

b Data from Smith et al. (2015).

### Enamel microstructure

**Figure 4:**
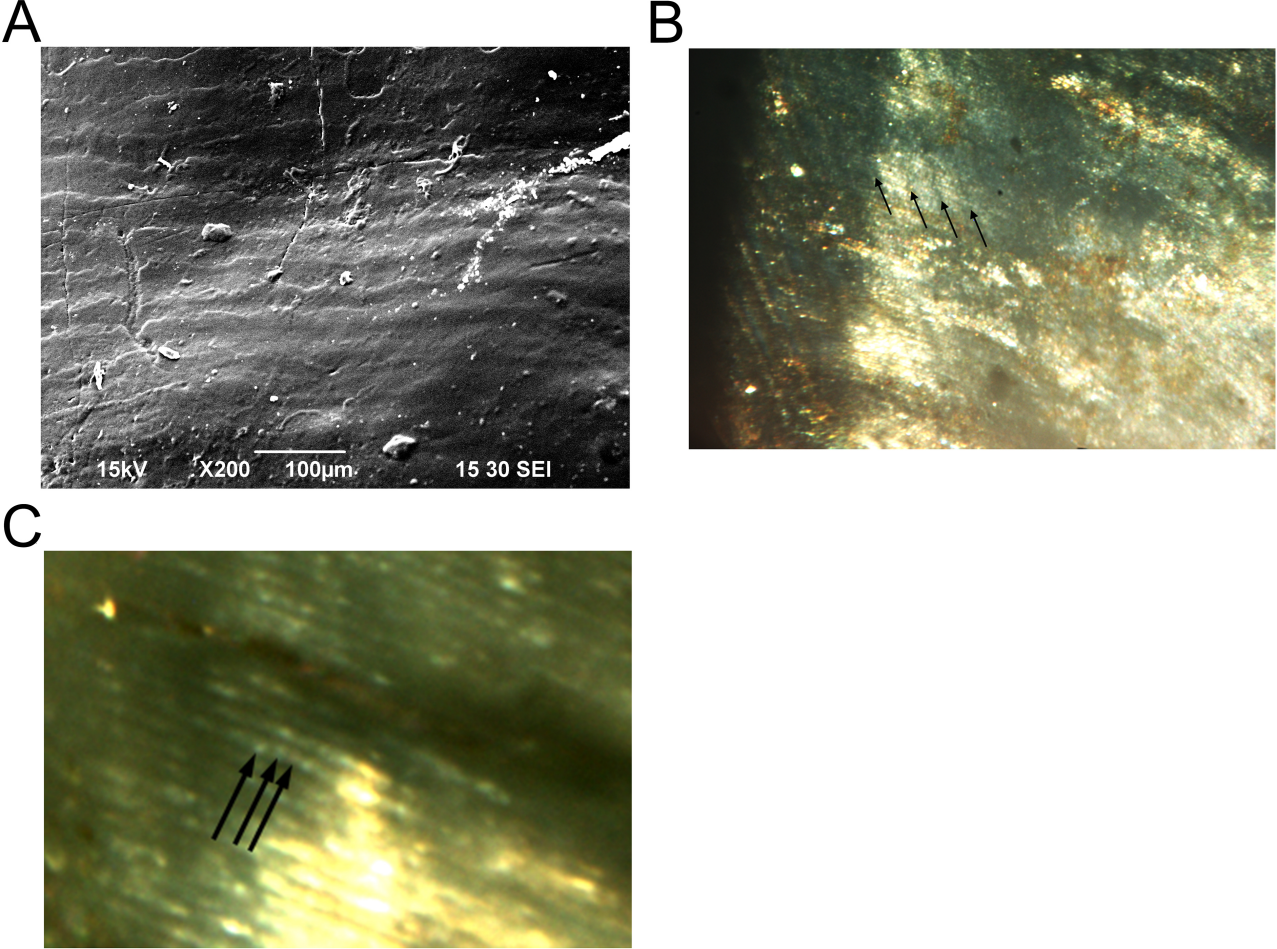
Enamel microstructure and microwear analyses of the HGT 500 specimen. (A) SEM image of the cervical portion of the molar demonstrating perikymata packing; (B) confocal microscopy (CM) image of the lateral aspect of the naturally broken enamel surface of the HGT 500 specimen demonstrating the shape of the striae of Retzius (arrows); (C) CM image of the same exposed enamel region as (B) with visible cross striations (arrows). Scale bars as indicated.

Analysis of the perikymata packing pattern along the cervical portion of the tooth revealed relatively packed perikymata (Fig. 4A). Enamel microstructural details (Hunter–Schreger bands, striae of Retzius shape, cross striations) were observed more clearly on the naturally broken surface. Hunter–Schreger bands appeared long and straight (Fig. 4B). Confocal microscopy revealed striae of Retzius in places along the lateral and cervical portion of the enamel but appeared faint at the occlusal enamel. Where visible, the overall shape of the striae tend to be short and not curving rapidly toward the occlusal portion of the enamel (Fig. 4B). Limited data were obtained on cross striation length as these were only visible in a few areas of the mid-to-outer lateral enamel. In those areas, cross striations measured on average ∼4.1 µm. Although the striae periodicity could not be clearly determined, a range of 7–9 days was obtained by measuring cross striations in the vicinity and applying this measurement to the distance between two adjacent striae, an approach similar to that described in Lacruz, Ramirez-Rozzi & Bromage (2006) (Figs. 4B and 4C).

## Discussion

The suite of micro- and macro- morphological features preserved on the HGT 500 specimen allows attribution to the Tribe Hominini (*sensu* Wood & Richmond, 2000) and is inconsistent with the genus *Paranthropus* in several key respects. The limits of the preserved anatomy, however, preclude a definitive generic or species attribution. The occlusal linear enamel thickness of ∼2.0 mm is beyond reported enamel thickness for large-bodied cercopithecoid primates (e.g., *Theropithecus*; Swindler & Beynon, 1993), at the lower end of values assigned to *P. robustus* (Grine, 1984) and most consistent with the reported range for *A. africanus* and early *Homo* (Table 2). The 7–9 day striae of Retzius periodicity is consistent with the 6–9 day range evinced by *Australopithecus* and early *Homo* specimens (australopiths: ∼7 days, early *Homo*: ∼9 days (Lacruz et al., 2008); *A. africanus*: ∼8.5 days, early South African *Homo*: just above 7 days (Smith et al., 2015)) to the exclusion of other large-bodied primates (Bromage et al., 2009). Long and straight Hunter–Schreger bands are visible on the naturally broken surface; however these features can be quite variable as they are affected by the plane of the examined section (Ramirez-Rozzi, 1998). The bands on the HGT 500 specimen are comparable to some of these features described for *Paranthropus* (Beynon & Wood, 1986; Ramirez-Rozzi, 1993), but are also seen in some specimens of east African *Australopithecus* and some early *Homo* (Beynon & Wood, 1986; Lacruz & Ramirez-Rozzi, 2010; Lacruz et al., 2012). Within the HGT 500 specimen, we observed tight packing of the perikymata on the cervical portion of the tooth. Tightly packed perikymata are observed in specimens assigned to *Australopithecus* and early *Homo* (Lacruz, Ramirez-Rozzi & Bromage, 2006) and differ from that described for *Paranthropus* molars where the perikymata are widely spaced (Dean et al., 1993). The portions of the striae of Retzius that are visible also suggest that the striae were not long; contrasting with the long striae described for East and South African *Paranthropus* species (Beynon & Wood, 1986; Ramirez-Rozzi, 1993; Lacruz, Ramirez-Rozzi & Bromage, 2006).

Irrespective of a more specific allocation of the specimen, the recovery of HGT 500 from the already primate-rich Haasgat locality is not unexpected given the taxonomically-diverse South African *Australopithecus* and *Homo* community proposed during the time period (between 2.3 and 1.95 Ma; but likely closer to 1.95 Ma) encompassed by the Haasgat *in situ* paleocave deposits. *Australopithecus africanus* is ubiquitous on the South African landscape from approximately 3.0–2.6 Ma (Makapansgat Limeworks and Taung) until ∼2.0 Ma (Sterkfontein Member 4; Herries et al., 2010; Herries & Adams, 2013), and the genus persists in the South African record later into the Pleistocene than in eastern Africa via *Australopithecus sediba* dated to approximately 1.98 Ma (Malapa; Pickering et al., 2011a). As such, if HGT 500 is *Australopithecus* then this is entirely in keeping with what we know about its temporal range and only slightly extends its geographic range to the north and east from Malapa. The oldest known fossils attributed to early *Homo* in South Africa are from the Swartkrans Member 1 deposits and date to between 2.3 and 1.8 Ma (Pickering et al., 2011b), although it has been suggested that the deposits date to between 2.0 and 1.8 Ma (and likely closer to 1.8 Ma; Herries & Adams, 2013). As Grine (2013) has also pointed out, it is perhaps unlikely that Swartkrans is as old as 2.3 Ma based on the age of *Australopithecus* bearing deposits at Sterkfontein, as there remains little evidence to suggest that early *Homo* and *Paranthropus* overlapped in the region contemporaneously with *Australopithecus.* At Sterkfontein the >2 Ma Member 4 deposits are generally considered to contain only *Australopithecus*. Although Kimbel & Rak (1993) have argued for the inclusion of Sts 19 in early *Homo*, this has been challenged by both Ahern (1998) and Lockwood & Tobias (2002). Early *Homo* fossils have also been recovered from Drimolen (Moggi-Cecchi et al., 2010), however the age of the deposits at the site remains only broadly established (between 2.0 and 1.4 Ma; Herries & Adams, 2013).

Ultimately, the significance of the HGT 500 specimen lies with the continued expansion of known hominin localities into the topographically and paleoecologically unique regions of South Africa outside the Bloubank Valley paleocave systems. Despite the limited geographic area encompassed by the Fossil Hominid Sites of South Africa World Heritage Site there are substantial differences in landscape topography and reconstructed mammalian paleocommunities between the southwestern Bloubank Valley sites (e.g., Sterkfontein, Swartkrans, Kromdraai, Bolt’s Farm, Cooper’s) and the few fossil localities, like Haasgat, that have been identified to the north or east of this well-sampled region (e.g., Gondolin, Gladysvale, Malapa, Motsetse, Hoogland). The northeastern World Heritage Site area is within the Skurweberg mountain range defined by sharp topographic relief due to differential erosion of the less chert-rich Monte Cristo dolomites in the southern region relative to the more chert-rich Eccles Formation dolomites in the northern region. This somewhat mirrors the higher topographic relief surrounding the Makapansgat site ∼250 km to the northwest of the Bloubank Valley sites. Faunal data from the Gondolin GD 2 deposits (Adams & Conroy, 2005; Adams, 2006) and Haasgat (Adams, 2012; Herries et al., 2014), that lie ∼4 km from each other, support the presence of a proto-Skurweberg range before 2 Ma, while cosmogenic nuclide exposure dating of quartz near Malapa (Dirks et al., 2010) also indicates substantial differential erosion of the region since the early Pleistocene that may have radically reshaped the topography from an upland plateau to the modern mountainous topography. Although there are similarities between the Haasgat and Gondolin faunal assemblages that underlies a regional paleoecological interpretation of locally rocky and broken terrain by 2 Ma, there are also substantial differences in the current faunal samples; ranging from significant metric differences between the Haasgat HGD and Gondolin GD 2 klipspringer (*Oreotragus* sp.) populations (Adams, 2012), to the proportions of cercopithecoids (with Gondolin GD 2 lacking any non-hominin primates; Adams & Conroy, 2005), and hominin representation (with Gondolin recording two isolated *ex situ* hominin teeth, only one of which has been specifically attributed to *Paranthropus robustus*; Menter et al., 1999; Grine et al., 2012; Herries & Adams, 2013). Establishing Haasgat as one of few hominin-bearing localities in this unique region affords more insight into the range of South African paleohabitats hominin populations occupied and the early Pleistocene paleocommunities that hominin populations interacted with; particularly critical in establishing the larger ecological context of the major hominin lineage transitions occurring ∼2 Ma.

## Conclusion

All of the South African sites from which these hominin species have been recovered are, for the most part, in an extremely constrained geographical range that would have presented similar adaptive pressures on early Pleistocene hominin populations. This is reflected in the considerable overlap across South African hominin species in stable isotope values (Lee-Thorp et al., 2010), morphological features associated with dietary adaptation such as enamel thickness (Smith et al., 2015) or dental crown metrics (Moggi-Cecchi et al., 2010), and retention of shared dental development patterns (Dean et al., 1993; Dean et al., 2001; Dean, 2010; Smith et al., 2015). These factors, when combined with the incomplete state of the HGT 500 specimen, contribute to the non-diagnostic nature of HGT 500 for taxonomic attribution. This first recovery of hominin material at Haasgat reinforces the potential for development of the ∼1.95 Ma site deposits to address outstanding paleoanthropological and paleontological research questions about hominin, primate, faunal and paleoecological variability outside the Bloubank Valley in the poorly-sampled northeastern Malmani dolomites that range between Malapa and Gondolin (Adams & Conroy, 2005; Herries et al., 2006; Adams et al., 2007; Berger et al., 2010; Adams, 2012; Grine et al., 2012; Adams, Kegley & Krigbaum, 2013; Gilbert et al., 2015).

## Additional Information and Declarations

### Data Availability

The following information was supplied regarding data availability: MorphoSource (www.morphosource.org), accession ID: M7737-8952; 10.17602/M2/M8952;
http://ezid.cdlib.org/id/doi:10.17602/M2/M8952.
